# In silico analysis of differentially expressed genesets in metastatic breast cancer identifies potential prognostic biomarkers

**DOI:** 10.1186/s12957-021-02301-7

**Published:** 2021-06-25

**Authors:** Jongchan Kim

**Affiliations:** Department of Life Sciences, Sogang University, 35 Baekbeom-ro, Mapo-gu, Seoul, 04107 Republic of Korea

**Keywords:** Breast cancer, Metastatic breast cancer, Prognosis, Oncomine, Gene ontology, Biomarkers

## Abstract

**Background:**

Identification of specific biological functions, pathways, and appropriate prognostic biomarkers is essential to accurately predict the clinical outcomes of and apply efficient treatment for breast cancer patients.

**Methods:**

To search for metastatic breast cancer-specific biological functions, pathways, and novel biomarkers in breast cancer, gene expression datasets of metastatic breast cancer were obtained from Oncomine, an online data mining platform. Over- and under-expressed genesets were collected and the differentially expressed genes were screened from four datasets with large sample sizes (N > 200). They were analyzed for gene ontology (GO), KEGG pathway, protein-protein interaction, and hub gene analyses using online bioinformatic tools (Enrichr, STRING, and Cytoscape) to find enriched functions and pathways in metastatic breast cancer. To identify novel prognostic biomarkers in breast cancer, differentially expressed genes were screened from the entire twelve datasets with any sample sizes and tested for expression correlation and survival analyses using online tools such as KM plotter and bc-GenExMiner.

**Results:**

Compared to non-metastatic breast cancer, 193 and 144 genes were differentially over- and under-expressed in metastatic breast cancer, respectively, and they were significantly enriched in regulating cell death, epidermal growth factor receptor signaling, and membrane and cytoskeletal structures according to the GO analyses. In addition, genes involved in progesterone- and estrogen-related signalings were enriched according to KEGG pathway analyses. Hub genes were identified via protein-protein interaction network analysis. Moreover, four differentially over-expressed (*CCNA2*, *CENPN*, *DEPDC1*, and *TTK*) and three differentially under-expressed genes (*ABAT*, *LRIG1*, and *PGR*) were further identified as novel biomarker candidate genes from the entire twelve datasets. Over- and under-expressed biomarker candidate genes were positively and negatively correlated with the aggressive and metastatic nature of breast cancer and were associated with poor and good prognosis of breast cancer patients, respectively.

**Conclusions:**

Transcriptome datasets of metastatic breast cancer obtained from Oncomine allow the identification of metastatic breast cancer-specific biological functions, pathways, and novel biomarkers to predict clinical outcomes of breast cancer patients. Further functional studies are needed to warrant validation of their roles as functional tumor-promoting or tumor-suppressing genes.

**Supplementary Information:**

The online version contains supplementary material available at 10.1186/s12957-021-02301-7.

## Background

World Health Organization reports that breast cancer is the most frequent female malignancy (www.who.int). Although conventional therapeutic strategies, including surgery, radiotherapy and chemotherapy, targeted therapies, and more recently immunotherapies [1, 2] dramatically prolonged the survival of breast cancer patients, the incidence and mortality rates of some subtypes continuously increase in recent years and the trend even varies depending on the race, age, or region [3, 4]. Identification of novel biomarkers in breast cancer is critical for accurate prognosis analysis and therapeutic efficacy prediction.

Stage IV breast cancers, in particular, are detrimental metastatic breast cancers (MBCs). MBCs are rarely curative, so their 5-year survival rate (26%) is much lower than localized cancer (99%) [5, 6]. Recently [7–14] and in the past, numbers of bioinformatic analyses have been conducted to identify key differentially expressed genes and enriched biological pathways or to evaluate the expression of a few specific genes in breast cancers, but such analysis using transcriptomes of MBCs has not been satisfactorily performed. The identification of biological functions and pathways enriched in MBCs is pivotal to search for appropriate treatment options that would minimize the adverse effects and increase the survival rates of this fatal disease.

ONCOMINE is a cancer microarray database and web-based data-mining platform containing 729 available datasets with 91,866 samples as of December 17th, 2020 (www.oncomine.org/) [15]. I searched for gene expression datasets generated with MBC patient samples and screened differentially over- and under-expressed genes. With the genesets, I attempted to analyze biological functions and pathways enriched in MBCs, to identify novel biomarker candidate genes positively and negatively correlated with the aggressive and metastatic nature of breast cancer and to validate their prognostic values in breast cancer. To do so, I conducted gene ontology (GO), Kyoto Encyclopedia of Genes and Genomes (KEGG) pathway analysis, protein-protein interaction (PPI) network analysis, hub gene identification, co-expression analysis, and Kaplan-Meier survival analyses with available online tools.

Ultimately, these analyses demonstrate that the identified genes may serve as potential prognostic biomarkers that accurately predict the clinical outcomes of breast cancer patients. The results also provide therapeutic implications that might be beneficial for treating metastatic breast cancer patients. Furthermore, the present study recapitulates the usefulness of Oncomine platform in identifying appropriate key pathways and biomarkers to suggest therapeutic opportunities and accurately predict the clinical outcomes of breast cancer patients.

## Methods

### Dataset acquisition

To obtain microarray datasets, the publicly available Oncomine data-mining platform (http://www.oncomine.org) was analyzed. Datasets that profiled metastatic breast cancers (MBCs) were retrieved using filters including “breast cancer” (cancer type) and “metastatic event status at three years” (Clinical Outcome). A total of fourteen datasets were available under these filters and only transcriptome datasets were chosen (two genomic DNA studies were excluded): Bos Breast (N > 200), Desmedt Breast (N < 200), Hatzis Breast (N > 200), Kao Breast (N > 200), Loi Breast (N < 200), Loi Breast 3 (N < 200), Minn Breast 2 (N < 200), Schmidt Breast (N < 200), Symmans Breast 2 (N < 200), Symmans Breast (N < 200), vandeVijver Breast (N > 200), and Vantveer Breast (N < 200).

### Determination of differentially expressed genesets

From four datasets with large sample sizes (N > 200), significantly over-expressed (fold change > 1) (DOE-L) or under-expressed (fold change < − 1) (DUE-L) genesets were selected based on their *P* values (*P* < 0.05) compared to the breast cancer patient samples with no metastatic events. 4797/2009 in Bos Breast, 3607/3564 in Hatzis Breast, 2375/2191 in Kao Breast, and 2350/2432 genes in vandeVijver Breast were significantly over-expressed/under-expressed, respectively. Using a Venn diagram drawing tool (http://bioinformatics.psb.ugent.be/webtools/Venn/), common genes were selected. In total, 193 and 144 genesets were differentially over-expressed (DOE-L) and under-expressed (DUE-L), respectively. These genesets were subjected to gene ontology, KEGG pathway, protein-protein interaction network analysis, and hub gene analyses to search for MBC-enriched genes, biological functions, and pathways.

To identify novel prognostic biomarkers, on the other hand, all twelve datasets with any sample sizes were analyzed. Differentially over-expressed (fold change > 1) or under-expressed (fold change < − 1) genesets with statistical significance (*P* < 0.05) were screened and examined. There was no single common gene found from all twelve datasets. However, four genes (*CCNA2*, *CENPN*, *TTK*, and *DEPDC1*) were differentially over-expressed (DOE-A) in eleven datasets (except Minn Breast 2) and one gene each was differentially under-expressed (DUE-A) in each of three groups of eleven datasets (the gene *ABAT* in the all twelve except Kao Breast, the gene *LRIG1* in the all twelve except Symmans Breast 2 and the gene *PGR* in the all twelve except Minn Breast 2).

### Gene ontology (GO) and KEGG pathway analyses

Differentially expressed (DOE-L and DUE-L) genes obtained from four breast cancer datasets with large sample numbers (N > 200) were subjected to gene ontology (GO) and KEGG pathway analyses for functional and characteristic classification of enriched genes. To do so, 337 genes including 193 DOE-L and 144 DUE-L genesets were entered and analyzed at Enrichr (https://amp.pharm.mssm.edu/Enrichr), an online analysis tool. Genes were classified into three GO categories; Biological Process, Molecular Function, and Cellular Component. KEGG (Kyoto Encyclopedia of Genes and Genomes) analysis for biological pathways was also conducted at Enrichr. The top ten GO terms and pathways were sorted according to their *P* values.

### Protein-protein interactions (PPIs) and hub protein identification

To examine the protein-protein interaction network within the differentially expressed genesets, I utilized the Search Tool for the Retrieval of Interacting Genes/Proteins (STRING). In total, 193 DOE-L and 144 DUE-L genesets were separately entered and their protein-protein interaction networks were analyzed. The networks were created, exported, and entered into Cytoscape, the network analysis/visualization tool to identify hub proteins from the complex networks. Among eleven “node ranking methods” [16], I analyzed the networks by Degree and both top ten hub proteins (hub_oe and hub_ue) were screened and ranked based on their number of interactors.

### Comparison of biomarker candidate gene expression between basal-like/triple-negative and other subtypes of breast cancers

Two online RNA-seq databases (Cancer Cell Line Encyclopedia (CCLE) for human breast cancer cell lines and bc-GenExMiner (version 4.3) for breast cancer patient samples) were used to compare the expression levels of four DOE-A and three DUE-A genes between basal-like/triple-negative and other subtypes of breast cancer. For CCLE, basal-like/triple-negative breast cancer (BL/TNBC) and luminal breast cancer cell lines were determined based on the literature [17–19]. For bc-GenExMiner, basal-like and TNBCs were determined by Prediction Analysis of Microarray 50 (PAM50) test and immunohistochemistry (IHC), respectively.

### Kaplan-Meier survival analyses

Survival tests including relapse-free survival (RFS), overall survival (OS), distant metastasis-free survival (DMFS), and post-progression survival (PPS) were performed using KM plotter at http://kmplot.com with Jetset best probe sets. MRFS was tested at http://bcgenex.centregauducheau.fr with all microarray datasets. All survivals were compared between the patients with high or low expression of each gene and the patient cohorts were split into two groups according to the median gene expression.

### Protocol registration

The research protocol used in the this study has been registered in PROSPERO database (registration #CRD42021247804).

### Statistical analysis

Statistical analyses were performed according to the pre-set analytic methods of each online tool. Two-tailed, unpaired *t*-tests were performed for comparing gene expression with CCLE dataset analysis following grouping the breast cancer cell lines into either luminal or BL/TNBC. *P* < 0.05 was considered statistically significant.

## Results

### Identification of differentially expressed genesets in metastatic breast cancers.

**Fig. 1:**
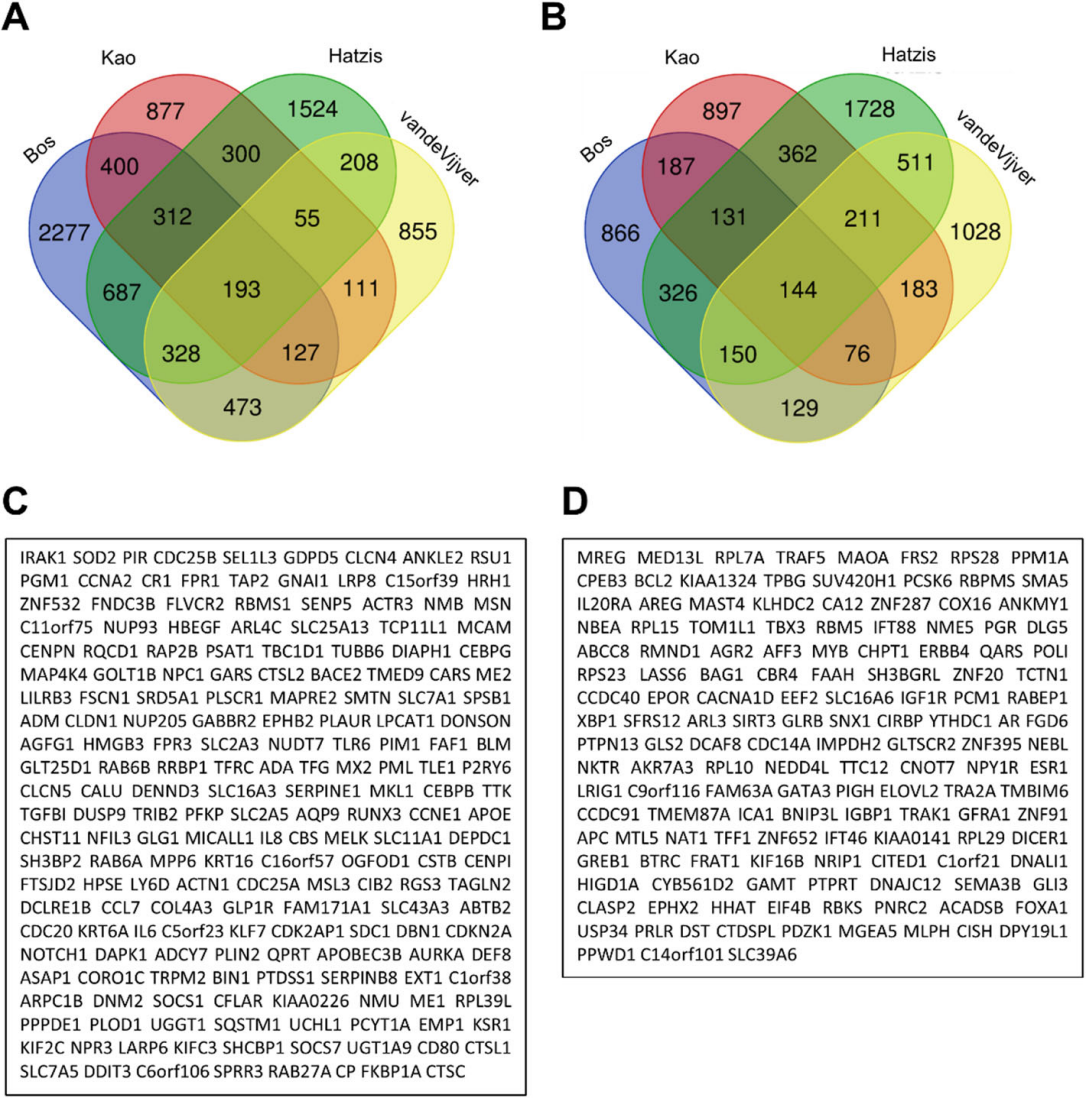
Identification of differentially over-expressed and under-expressed genes in metastatic breast cancer. **A**, B Venn diagrams to screen significantly (*P* < 0.05) over-expressed (fold change > 1) (**A**) and under-expressed genes (fold change < − 1) (**B**) in metastatic breast cancer (MBC). **C**,**D** 193 and 144 differentially over-expressed and under-expressed genes in MBCs are listed, respectively

We identified differentially over-expressed (DOE-L) and under-expressed (DUE-L) genes in metastatic breast cancer (MBC) by utilizing the Oncomine database (Tables S1 and S2). A total of 193 DOE-L and 144 DUE-L genes were selected (Fig. 1) as described in Methods.

### Functional and characteristic classification of enriched genes in metastatic breast cancer.

**Fig. 2:**
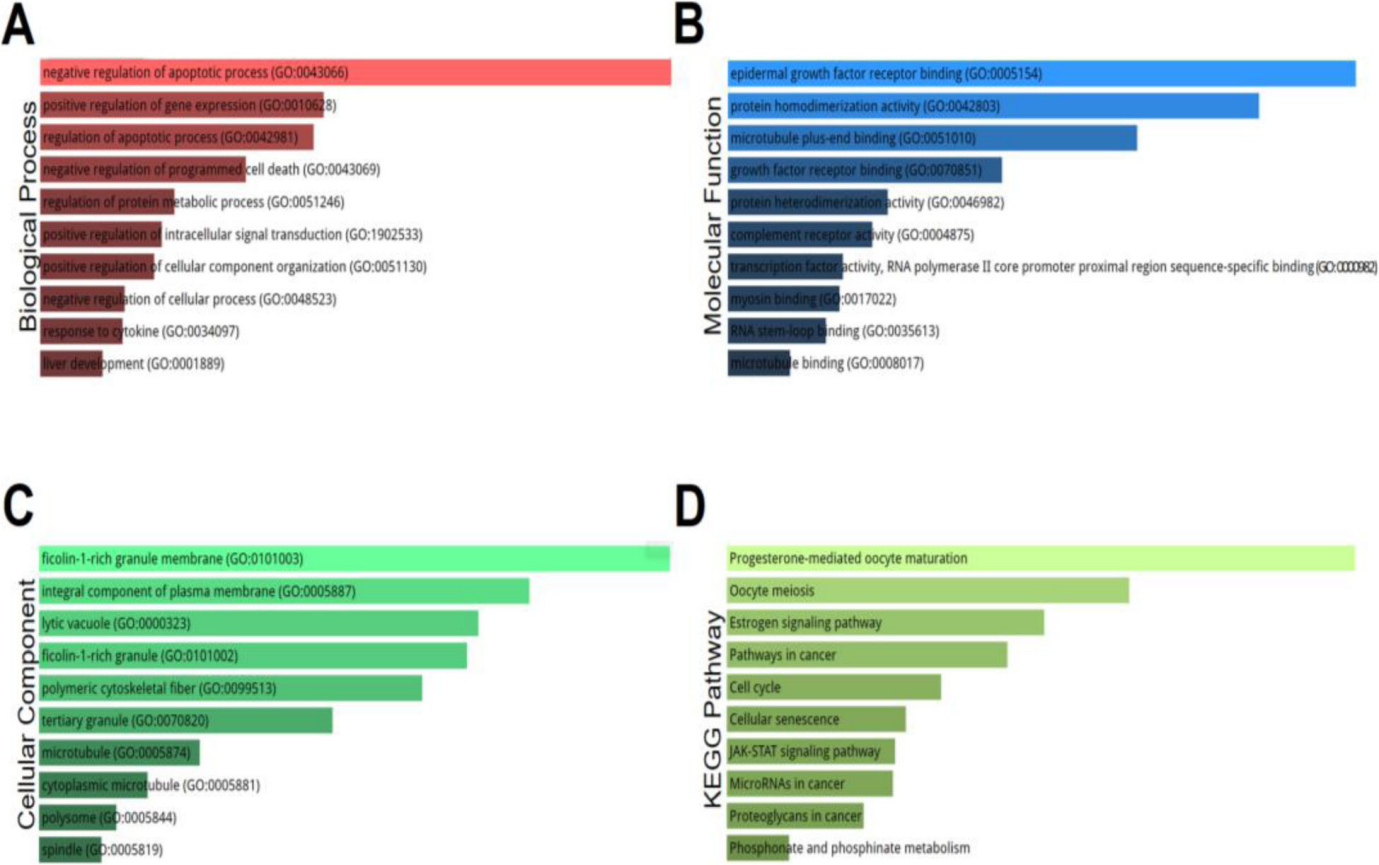
Gene ontology (GO) and KEGG pathway analyses in metastatic breast cancer. **A**–**D** Using 337 genes including 193 differentially over-expressed and 144 under-expressed genes, functional enrichment in metastatic breast cancer was examined based on the three GO categories (biological process (**A**), molecular function (**B**), and cellular component (**C**)) and on the KEGG pathway (**D**) at Enrichr. Genes are ranked and listed according to the statistical significance (*P* values)

To analyze the functional enrichment of the differentially expressed genes in MBCs, I examined gene ontology (GO) and KEGG (Kyoto Encyclopedia of Genes and Genomes) pathway analysis using 337 differentially expressed genes (193 DOE-L and 144 DUE-L genes). They were classified into three GO categories including biological process (BP), molecular function (MF), and cellular component (CC). For BP, genes are significantly enriched in the GO terms including negative regulation of the apoptotic process, positive regulation of gene expression, regulation of the apoptotic process, negative regulation of programmed cell death, and regulation of protein metabolic process (Fig. 2A). For MF, genes are significantly enriched in the GO terms including epidermal growth factor receptor binding, protein homodimerization activity, microtubule plus-end binding, growth factor receptor binding, and protein heterodimerization activity (Fig. 2B). For CC, genes are significantly enriched in the GO terms including ficolin-1-rich granule membrane, an integral component of the plasma membrane, lytic vacuole, ficolin-1-rich granule, and polymeric cytoskeletal fiber (Fig. 2C). Therefore, these results suggest that genes regulating cell death, gene expression, protein metabolism, signal transduction, and protein-protein binding are significantly enriched in MBCs. Also, KEGG pathway analysis demonstrates that genes involved in progesterone-mediated oocyte maturation, oocyte meiosis, estrogen signaling pathway, pathways in cancer, and cell cycle are also significantly enriched in MBCs (Fig. 2D).

### Interactome networks of the differentially expressed genes and identification of hub genes in metastatic breast cancer

**Fig. 3:**
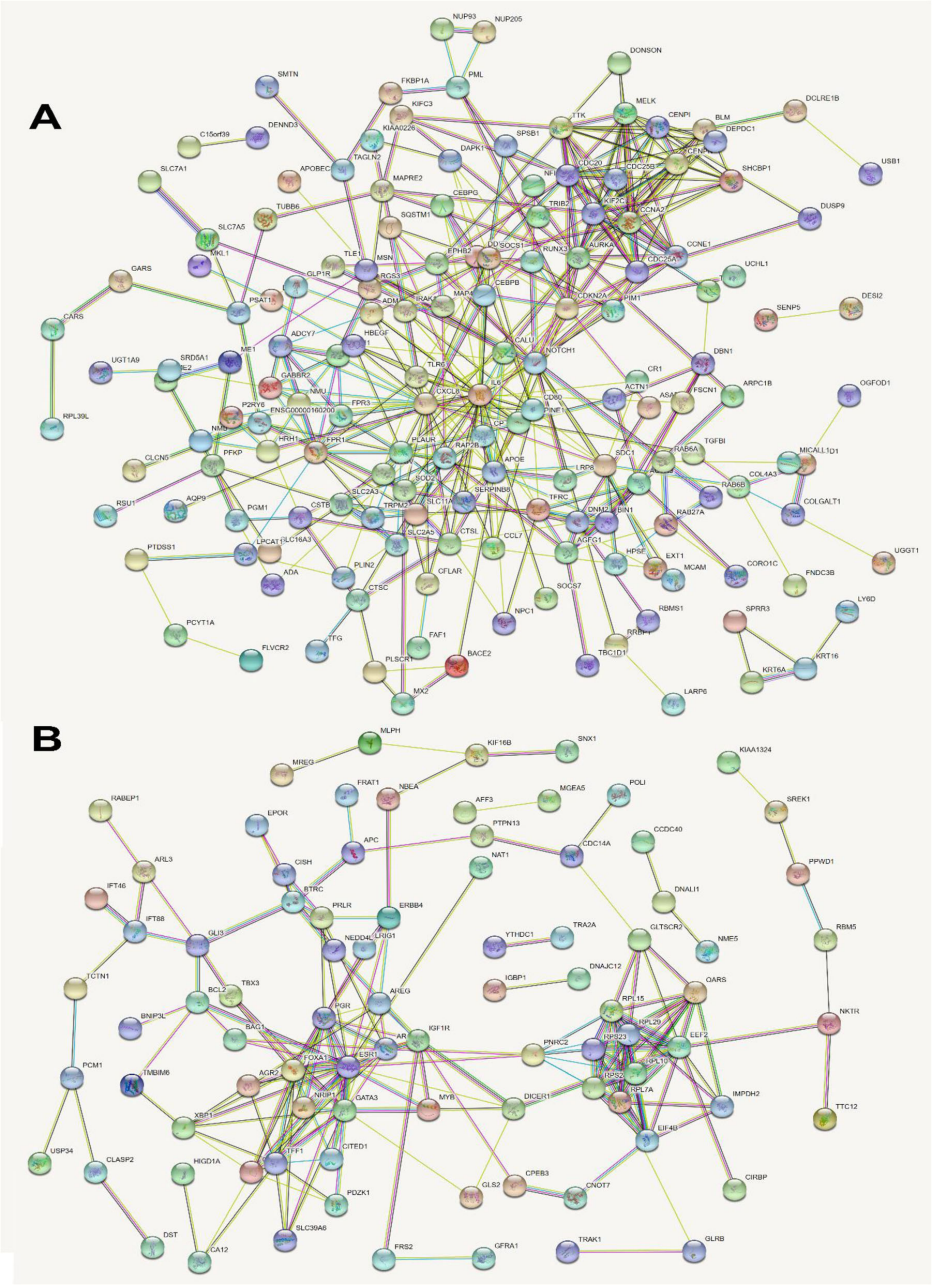
Protein-protein interaction networks of differentially expressed genes in metastatic breast cancer. **A** Protein-protein interaction (PPI) among 193 differentially over-expressed genes was examined to analyze the functional protein association network in metastatic breast cancer using STRING, an online tool. In total, 192 nodes (disconnected nodes are hidden) and 407 edges are presented. PPI enrichment *P* value is 1.11 × 10^−16^, which implies this network has significantly more interactions than a network with a randomly chosen set of proteins. **B** Protein-protein interaction (PPI) among 144 differentially under-expressed genes was examined to analyze the functional protein association network in metastatic breast cancer using STRING. In total, 143 nodes (disconnected nodes are hidden) and 190 edges are presented. PPI enrichment *P* value is 6.99 × 10^−14^, which implies this network has significantly more interactions than a network with a randomly chosen set of proteins. Circles and lines in **A** and **B** indicate genes and interactions, respectively. The line colors indicate the types of interaction evidence.

Protein-protein interaction (PPI) provides insights into molecular function and diseases including cancer [20]. To explore PPI networks of the differentially expressed genes in MBCs, I utilized STRING, an online protein-protein interaction prediction tool, which visualizes potential interaction networks based on experimentally proven interaction data and computational prediction [21]. DOE-L (Fig. 3A) and DUE-L genes (Fig. 3B) were separately subjected to PPI analysis. In total, 192 nodes and 407 edges from DOE-L genes and 143 nodes and 190 edges from DUE-L genes were predicted after excluding disconnected nodes. Of note, their PPIs were predicted significantly more than those of a randomly chosen set of proteins.

**Fig. 4:**
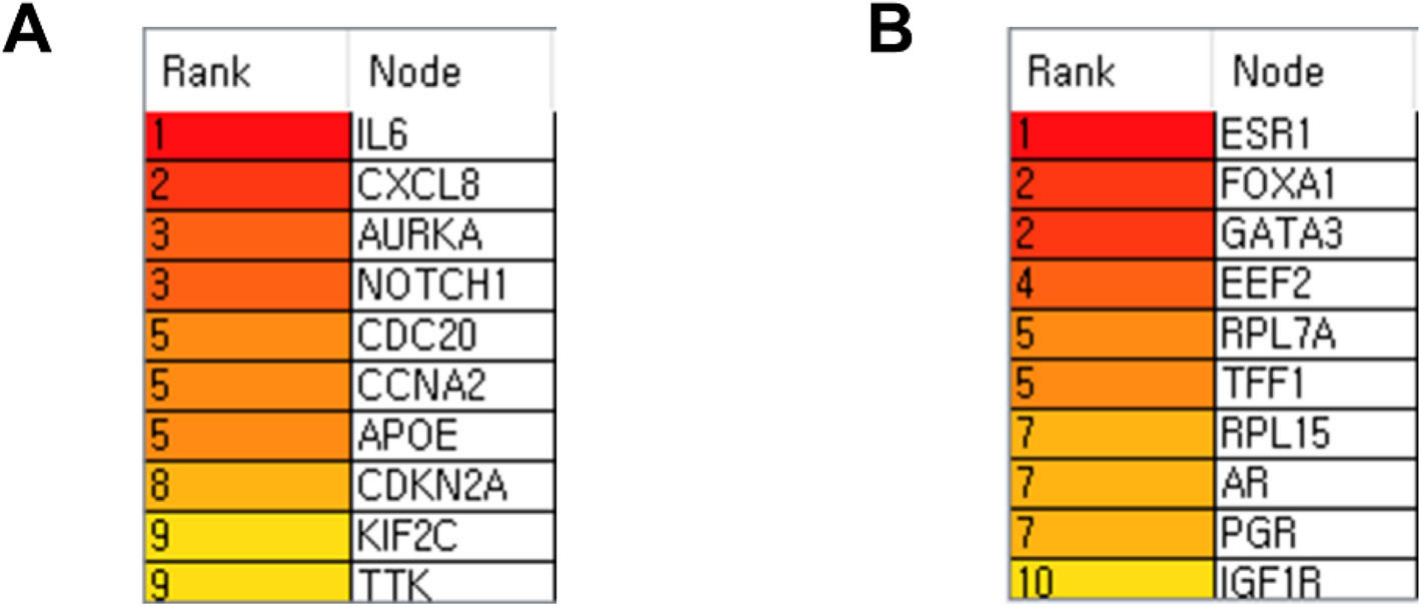
Identification of hub genes in PPI networks. **A,B** The PPI networks among the differentially over-expressed (**A**) and under-expressed genes (**B**) were exported separately and entered into Cytoscape, the network analysis tool. The top ten hub genes (**A**, hub_oe; **B**, hub_ue) are ranked according to their number of interactors

To identify hub genes based on the PPI networks, I exported each network and examined them according to the degree of connectivity (DC) using Cytoscape software. In the DOE-L geneset, *IL6* (DC = 32), *CXCL8* (DC = 27), *AURKA/NOTCH1* (DC = 21), *CDC20/CCNA2/APOE* (DC = 17), *CDKN2A* (DC = 16), and *KIF2C/TTK* (DC = 15) were ranked as top ten hub genes (hub_oe) (Fig. 4A). In addition, *ESR1* (DC = 22), *FOXA1/GATA3* (DC = 14), *EEF2* (DC = 13), *RPL7A/TFF1* (DC = 12), *RPL15/AR/PGR* (DC = 11), and *IGF1R* (DC = 10) in DUE-L genes were ranked as top ten hub genes (hub_ue) (Fig. 4B).

### Identification of novel biomarker candidate genes for breast cancer

As shown in Table 1, four (*CCNA2*, *CENPN*, *DEPDC1*, and *TTK*; DOE-A) and three genes (*ABAT*, *LRIG1*, and *PGR*; DUE-A) were identified as differentially over- and under-expressed genes, respectively, as described in “Methods” and they were selected as novel biomarker candidate genes for breast cancer and were subjected to the subsequent analyses.

**Table 1:** Potential biomarker candidate genes in metastatic breast cancer gene expression datasets

**Potential biomarker candidate genes (over-expressed; DOE-A)**	**Relevant 11 studies**
*CCNA2*	Bos Breast, Desmedt Breast, Hatzis Breast, Kao Breast, Loi Breast, Loi Breast 3, Schmidt Breast, Symmans Breast 2, Symmans Breast, vandeVijver Breast, Vantveer Breast
*CENPN*	
*DEPDC1*	
*TTK*	
**Potential biomarker candidate genes (under-expressed; DUE-A)**	**Relevant 11 studies**
*ABAT*	Bos Breast, Desmedt Breast, Hatzis Breast, Loi Breast, Loi Breast 3, Minn Breast 2, Schmidt Breast, Symmans Breast 2, Symmans Breast, vandeVijver Breast, and Vantveer Breast
*LRIG1*	Bos Breast, Desmedt Breast, Hatzis Breast, Kao Breast, Loi Breast, Loi Breast 3, Minn Breast 2, Schmidt Breast, Symmans Breast, vandeVijver Breast, and Vantveer Breast
*PGR*	Bos Breast, Desmedt Breast, Hatzis Breast, Kao Breast, Loi Breast, Loi Breast 3, Schmidt Breast, Symmans Breast 2, Symmans Breast, vandeVijver Breast, and Vantveer Breast

### Identification of PPI hub genes co-expressed with potential biomarker candidates

I attempted to find PPI hub genes (hub_oe and hub_ue) the most significantly and positively co-expressed with four DOE-A and three DUE-A novel biomarker candidate genes, respectively. Among the top ten hub_oe genes (Fig. 4A), *KIF2C* was the only gene that is the most significantly (*P* < 0.0001) and positively co-expressed with all four potential biomarker candidate genes (*AURKA* (r = 0.75) was co-expressed as positively as *KIF2C* (r = 0.75) with *CENPN*) (Fig. S1A). Among the top ten hub_ue genes (Fig. 4B), *ESR1* was the only gene that is the most significantly (*P* < 0.0001) and positively co-expressed with all three potential biomarker candidate genes (*FOXA1* (r = 0.63) was co-expressed as positively as *ESR1* (r = 0.63) with *LRIG1*) (Fig. S1B)

### Examination of the expression correlation of potential biomarker candidate genes with the aggressive and metastatic nature of breast cancer

**Fig. 5:**
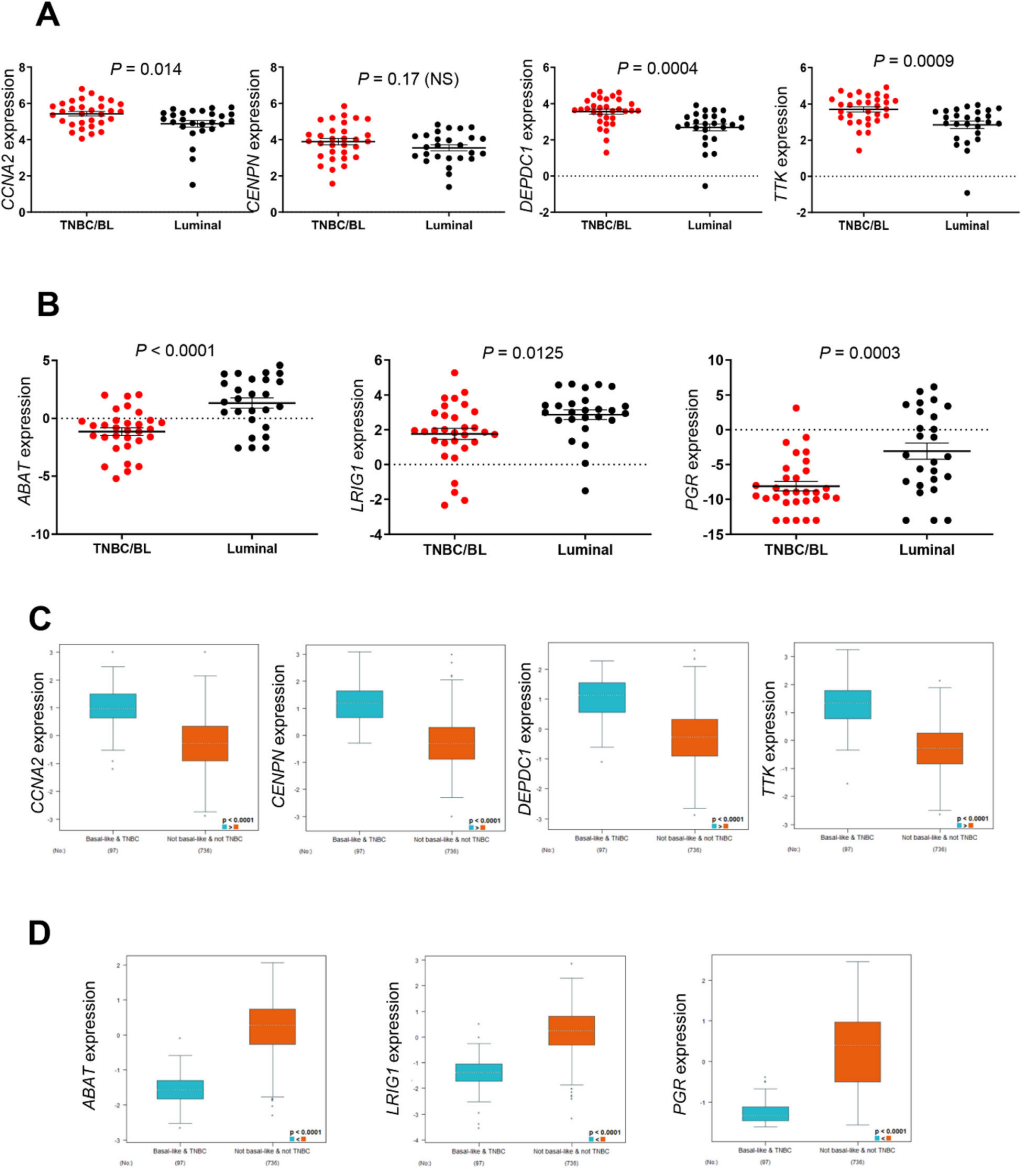
Comparison of mRNA expression of four DOE-A and three DUE-A potential biomarker candidate genes between basal-like and/or triple-negative breast cancer and other subtypes of breast cancer. **A** RNA-seq data of four DOE-A genes were obtained from the Cancer Cell Line Encyclopedia (CCLE) and analyzed. N = 31 in BL/TNBC and N = 26 in luminal type cell lines. **B** RNA-seq data of three DUE-A genes were obtained from the Cancer Cell Line Encyclopedia (CCLE) were analyzed. N = 31 in BL/TNBC and N = 26 in luminal type cell lines. **C** RNA-seq data of four DOE-A genes from The Cancer Genome Atlas (TCGA) [58] were analyzed at bc-GenExMiner v4.3. N = 97 in BL/TNBC and N = 736 in non-BL/TNBC type breast cancer patient samples. **D** RNA-seq data of three DUE-A genes from The Cancer Genome Atlas (TCGA) [58] were analyzed at bc-GenExMiner v4.3. N = 97 in BL/TNBC and N = 736 in non-BL/TNBC type breast cancer patient samples. Statistical significances in **A** and **B** were determined by unpaired *t*-tests and those in **C** and **D** were determined by the pre-set analytic method of bc-GenExMiner

Basal-like (BL) and/or triple-negative breast cancers (TNBCs) are considered an aggressive and highly metastatic subtype of breast cancer often associated with poor clinical outcomes [22–27]. To examine the expression correlation of the potential biomarker candidate genes (DOE-A and DUE-A) with the aggressive and metastatic nature of breast cancer, I compared their expression levels in BL/TNBCs with those in other breast cancer subtypes. First, I extracted RNA-seq expression data of human breast cancer cell lines from the Cancer Cell Line Encyclopedia (CCLE). A total of 57 human breast cancer cell lines have expression information in the database and their subtypes were determined based on previous reports [17–19]. Among them, 26 are luminal and 31 are BL/TNBCs. The expression levels of *CCNA2*, *DEPDC1*, and *TTK* (DOE-A) were significantly higher in BL/TNBCs than in luminal breast cancer cell lines (Fig. 5A). The expression of all three DUE-A genes, on the other hand, was significantly lower in BL/TNBC cell lines, compared to luminal breast cancer cell lines (Fig. 5B).

Next, I chose to further investigate whether this correlation in cell lines could also be applied to human breast cancer patient samples. Using an online tool called bc-GenExMiner (version 4.3), I compared the gene expression between BL/TNBCs and other subtypes of human breast cancers. Consistent with cell line analysis, all four DOE-A genes were expressed significantly more (Fig. 5C) and all three DUE-A genes were expressed significantly less in BL/TNBCs than in non-BL and non-TNBCs (Fig. 5D). Together, the results in Fig. 5 strongly demonstrate that seven potential biomarker candidate genes (DOE-A and DUE-A) are positively and negatively correlated with the aggressive and metastatic nature of breast cancer, respectively.

Additionally, I examined two of the most significantly co-expressed hub genes (*KIF2C* and *ESR1*) shown in Fig. S1 and found that *KIF2C* and *ESR1* were significantly up- and downregulated in BL/TNBCs, respectively, compared to luminal breast cancer cell lines (Figs. S2A and S2B). Moreover, in human breast cancer patient samples, the result was consistent (Figs. S2C and S2D). The data suggest that *KIF2C* and *ESR1*, two co-expressed hub genes, are also positively and negatively correlated with the aggressive and metastatic nature of breast cancer, respectively.

### Examination of prognostic values of the biomarker candidate genes in breast cancer patients

**Fig. 6:**
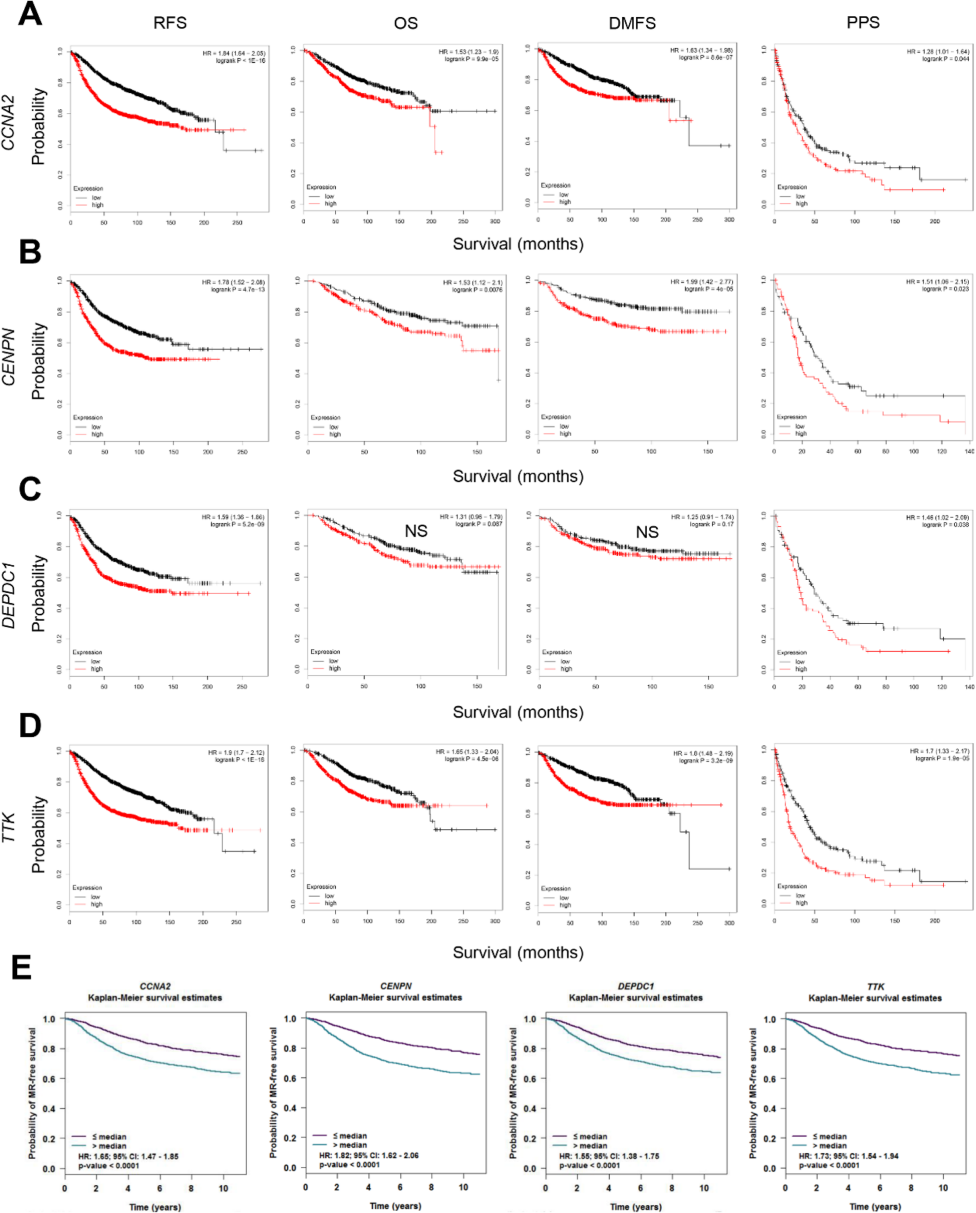
Correlation between the expression levels of potential prognostic biomarkers (DOE-A genes) and patient survival. **A–D** Relapse-free, overall, distant metastasis-free, and post-progression survival of four DOE-A genes (*CCNA2* in **A**; *CENPN* in **B**; *DEPDC1* in **C**; *TTK* in **D**) were stratified by the expression levels of each gene (low or high). Expression data were analyzed using KM plotter (http://kmplot.com/). JetSet best probes were selected and patients (for *CCNA2*, N = 3951 in RFS, = 1402 in OS, = 1746 in DMFS and = 414 in PPS; for *CENPN*, N = 1764 in RFS, = 626 in OS, = 664 in DMFS and = 173 in PPS; for *DEPDC1*, N = 1764 in = RFS, = 626 in OS, = 664 in DMFS and = 173 in PPS; for *TTK*, N = 3951 in RFS, = 1402 in OS, = 1746 in DMFS and = 414 in PPS) were split by median expression. NS, not significant. **E** Metastatic relapse-free survival of four DOE-A genes were stratified by the expression levels of each gene (low or high). Microarray expression data were analyzed using bc-GenExMiner v4.3 (http://bcgenex.centregauducheau.fr/). Patients (for *CCNA2* and *TTK*, N = 4533; for *CENPN* and *DEPDC1*, N = 4359) were split by median expression. Statistical analyses were performed by pre-set analytic methods. HRs (hazardous ratios) and 95% CIs (confidence intervals) are indicated

**Fig. 7:**
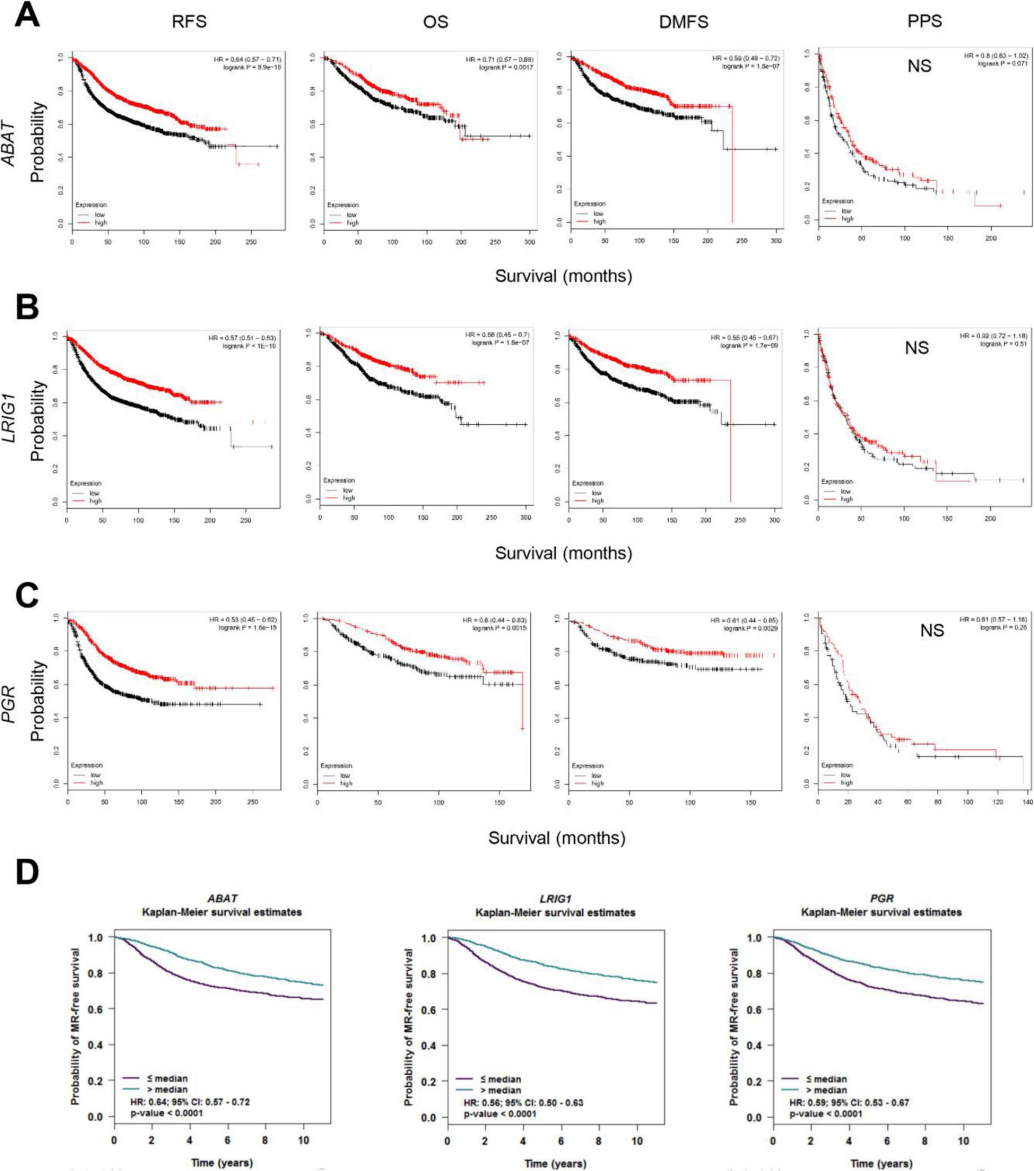
Correlation between the expression levels of potential prognostic biomarkers (DUE-A genes) and patient survivals. **A–C** Relapse-free, overall, distant metastasis-free, and post-progression survival of three DUE-A genes (*ABAT* in **A**; *LRIG1* in **B**; *PGR* in **C**) were stratified by the expression levels of each gene (low or high). Expression data were analyzed using KM plotter (http://kmplot.com/). JetSet best probes were selected and patients (for *ABAT and LRIG1*, N = 3951 in RFS, = 1402 in OS, = 1746 in DMFS and = 414 in PPS; for *PGR*, N = 1764 in RFS, = 626 in OS, = 664 in DMFS and = 173 in PPS) were split by median expression. NS, not significant. **D** Metastatic relapse-free survival of three DUE-A genes was stratified by the expression levels of each gene (low or high). Microarray expression data were analyzed by bc-GenExMiner v4.3 (http://bcgenex.centregauducheau.fr/). Patients (for *ABAT*, *LRIG1*, and *PGR*, N = 4434) were split by median expression. Statistical analyses were performed by pre-set analytic methods. HRs (hazardous ratios) and 95% CIs (confidence intervals) are indicated

To examine the prognostic values of four DOE-A and three DUE-A potential biomarkers in predicting breast cancer patient survival, I explored the correlation between their expression levels and the patients’ clinical outcomes. For DOE-A genes, high levels of *CCNA2*, *CENPN*, and *TTK* expression were significantly associated with poor prognosis in all four available patient survivals when analyzed with KM plotter (RFS, relapse-free survival; OS, overall survival; DMFS, distant metastasis-free survival; PPS, post-progression survival). High expression of *DEPDC1*, another DOE-A gene, was significantly associated with poor prognosis only in RFS and PPS **(**Fig. 6A–D). Besides, high levels of all four DOE-A gene expressions were significantly correlated with metastatic relapse-free survival (MRFS) when analyzed with bc-GenExMiner (version 4.3) (Fig. 6E). For DUE-A genes, on the other hand, high levels of all three DUE-A gene expression were significantly associated with good patient RFS, OS, DMFS (except PPS) (Fig. 7A–C) and MRFS (Fig. 7D). I also examined two of the most significantly co-expressed hub genes, *KIF2C* and *ESR1*, and found that they were also significantly associated with poor and good clinical outcomes, respectively, in all five survival analyses (Fig. S3).

## Discussion

Because of the limitations in the classical TNM staging system, The American Joint Committee on Cancer (AJCC) 8th edition added biological factors including estrogen and progesterone receptor expression and human epidermal growth factor 2 status for clinical prognostic staging in combination with the TNM staging [28]. Furthermore, when available, the use of multigene expression assays is recommended as stage modifiers [28]. By comparing the multigene assay panels recommended in AJCC 8th edition, I found that three biomarker genes (*ESR1*, *PGR*, and *KIF2C*) were already included in at least one of the panels and the rest six biomarker genes (*CCNA2*, *CENPN*, *DEPDC1*, *TTK*, *ABAT*, and *LRIG1*) were not included in any of them. This suggests that the present study applied reliable analytic methods that could reproduce the prognostic value of some biomarkers as well as present meaningful novel prognostic biomarkers. Each multigene panel is, however, limited to use only in patients with specific stages and pathology, which implicates that the biomarker genes identified in the present study need additional validations to confirm their proper utility in the particular patient groups based on the stages and pathology.

*CCNA2* encodes Cyclin A2 which functions as a cell cycle regulator and its expression is elevated in many human cancers. Moreover, *CCNA2* gene dysregulation is shown to be associated with poor prognosis [29–31]. *CENPN* encodes Centromere Protein N, which is important for the assembly of a multi-protein complex called kinetochore [32]. *DEPDC1* encodes DEP domain containing 1 protein, which has been shown to act as a transcription regulator by forming a complex with ZNF224, a member of the Krueppel C2H2-type zinc-finger protein family [33]. *TTK* encodes a dual-specificity protein kinase that can phosphorylate tyrosine and serine/threonine (threonine tyrosine kinase) and has crucial roles in regulating the spindle assembly checkpoint [34]. It is often overexpressed in breast tumors [35] and confers radioresistance [36]. *ABAT* encodes 4-aminobutyrate aminotransferase, which metabolizes GABA (γ-aminobutyric acid), a neurotransmitter. This gene expression is downregulated in inflammatory breast cancer and low expression of *ABAT* is correlated with a poor tamoxifen treatment outcome [37]. Moreover, it suppresses breast cancer metastasis [38]. *LRIG1* encodes a protein that negatively regulates epidermal growth factor receptor signaling, and its tumor-suppressive effects in cancer have been demonstrated [39–43]. *PGR* encodes the progesterone receptor, a member of the steroid receptor superfamily. Its expression is higher in luminal type A breast cancer than other aggressive breast cancer subtypes [44] and studies have demonstrated that progesterone receptor-positive (PR+) breast cancers are associated with better prognosis [45–47]. Furthermore, *KIF2C* encodes a kinesin-like microtubule-dependent motor protein, which depolymerizes microtubules and promotes chromosomal segregation [48, 49]. Its overexpression has been observed in human breast cancer cases and cell lines [50, 51]. *ESR1* encodes estrogen receptor α, a hormone receptor whose transcription activity is regulated by estrogen binding. Patients with estrogen receptor α positive (ERα+) breast tumors have demonstrated better survival and later recurrence than those with ERα- breast tumors [52–54] (Table 2).

**Table 2:** Summary of nine selected genes and their roles as prognostic biomarkers in breast cancer.

Gene symbols	Gene names	Prognostic value in breast cancer with high expression	Cellular/biological roles
*CCNA2*	Cyclin A2	Poor	Cell cycle regulator
*CENPN*	Centromere protein N	Poor	Regulates kinetochore assembly
*DEPDC1*	DEP domain containing 1	Poor	Transcription regulator
*TTK*	TTK protein kinase	Poor	Regulates the spindle assembly checkpoint
*KIF2C*	Kinesin family member 2C	Poor	Microtubule-dependent motor protein
*ABAT*	4-Aminobutyrate aminotransferase	Good	Metabolizes GABA
*LRIG1*	Leucine-rich repeats and immunoglobulin-like domains 1	Good	Negatively regulates EGFR signaling
*PGR*	Progesterone receptor	Good	Hormone receptor
*ESR1*	Estrogen Receptor 1	Good	Hormone receptor

Overall, it is interesting to note that cell cycle-related genes (*CCNA2*, *CENPN*, *TTK*, and *KIF2C*) and hormone signaling-related genes (*ABAT*, *PGR*, and *ESR1*) were differentially over- and under-expressed in the metastatic breast cancers, respectively. They were also predominantly associated with poor and good clinical outcomes, respectively. The results suggest that targeting cell cycle regulators may but hormonal therapy may not be beneficial for metastatic breast cancer patients, in general, although an individual patient may respond differently. Indeed, cell cycle inhibitors such as CDK4/6i (inhibitor of the cyclin-dependent kinases 4 and 6) have been approved and used for metastatic breast cancer patients either alone or in a combinational therapy [55].

In addition, I attempted to identify functional, biological, molecular, and cellular processes specifically altered in metastatic human breast cancers (MBCs). Differentially expressed genes in MBCs are mostly involved in regulating cell death, epidermal growth factor receptor signaling, and membrane and cytoskeletal structures, and are also enriched in biological pathways such as progesterone- and estrogen-related signaling. In fact, EGF receptor inhibition often fails in the treatment of metastatic breast cancer potentially due to the “paradoxical” anti-proliferative and anti-metastatic function of EGF receptor signaling [56], which implicates that EGF receptor inhibitors should be used with caution in metastatic breast cancer. Moreover, cancer metastasis and chemoresistance are demonstrated as a linked phenotype [57], which implies that chemotherapy-induced cell death signaling is fundamentally altered in metastatic breast cancer.

Although I demonstrated that the expression levels of potential biomarkers are positively/negatively correlated with the aggressive and metastatic nature of breast cancer and are associated with clinical outcomes of breast cancer patients, their molecular functions except for *CCNA2*, *PGR*, and *ESR1* have not been experimentally elucidated in breast carcinogenesis. Future functional validation is needed to warrant their potential values as breast cancer biomarkers as well as tumor-promoting or tumor-suppressing molecules. Also, the present study proves the usefulness of Oncomine platform to identify enriched pathways and potential prognostic biomarkers to predict beneficial treatment options for and the clinical outcomes of breast cancer.

## Conclusions

In the present study, I delineated biological functions and pathways specifically enriched in metastatic breast cancer and demonstrated that *CCNA2*, *CENPN*, *DEPDC1*, *TTK*, *ABAT*, *LRIG1*, *PGR*, *KIF2C*, and *ESR1* may serve as biomarkers to predict clinical outcomes of breast cancer patients. Pathway analysis suggests which therapeutic opportunities, in general, may or may not potentially be beneficial to the treatment of metastatic breast cancers. Additionally, the present study demonstrates the usefulness of Oncomine data-mining platform. Further functional studies are needed to warrant validation of the roles of selected genes as functional tumor-promoting or tumor-suppressing molecules.

## Supplementary information

**Additional file 1: **Supplementary Figure S1. *KIF2C* and *ESR1* are hub genes the most significantly co-expressed with the potential biomarker candidate genes. (A and B) Shown are Pearson's pairwise correlation plots of RNA-seq gene expression between four DOE-A (A) or three DUE-A genes (B) and their most significantly co-expressed hub genes identified from PPI networks. Statistical analyses were performed by the pre-set analytic method of bc-GenExMiner. Supplementary Figure S2. Comparison of mRNA expression of the two most significantly co-expressed hub genes (*KIF2C* and *ESR1*) between basal-like or triple-negative breast cancer and other subtypes of breast cancer. (A and B) RNA-seq data of *KIF2C* and *ESR1* were obtained from the Cancer Cell Line Encyclopedia (CCLE) and analyzed. N = 31 in BL/TNBC and N = 26 in luminal type cell lines. (C and D) RNA-seq data of *KIF2C* and *ESR1* from The Cancer Genome Atlas (TCGA) [58] were analyzed at bc-GenExMiner v4.3. N = 97 in BL/TNBC and N = 736 in non-BL/TNBC type breast cancer patient samples. Statistical significance in A and B was determined by unpaired *t*-tests and those in C and D were determined by the pre-set analytic method of bc-GenExMiner. Supplementary Figure S3. Correlation between the expression levels of two co-expressed hub genes (*KIF2C* and *ESR1*) and patient survivals. (A and B) Relapse-free, overall, distant metastasis-free, and post-progression survival of two co-expressed hub genes (*KIF2C* in (A); *ESR1* in (B)) were stratified by the expression levels of each gene (low or high). Expression data were analyzed by KM plotter (http://kmplot.com/). JetSet best probes were selected and patients (for *KIF2C*, N = 3951 in RFS, = 1402 in OS, = 1746 in DMFS and = 414 in PPS; for *ESR1*, N = 3951 in RFS, = 1402 in OS, = 1746 in DMFS and = 414 in PPS) were split by median expression. (C) Metastatic relapse-free survival of *KIF2C* and *ESR1* was stratified by the expression levels of each gene (low or high). Microarray expression data were analyzed by bc-GenExMiner v4.3 (http://bcgenex.centregauducheau.fr/). Patients (*KIF2C*, N = 4533; *ESR1*, N = 4785) were split by median expression. Statistical analyses were performed by pre-set analytic methods. HRs (hazardous ratios) and 95% CIs (confidence intervals) are indicated.

**Additional file 2:.** Table S1. Twelve raw datasets with over-expressed genes.

**Additional file 3:.** Table S2. Twelve raw datasets with under-expressed genes.

## Data Availability

The gene expression datasets are available at Oncomine.org.

## Abbreviations

MBC: Metastatic breast cancer
GO: Gene ontology
KEGG: Kyoto Encyclopedia of Genes and Genomes
PPI: Protein-protein interaction
DOE-L / DUE-L: Differentially over- or under-expressed genesets from the four datasets with large patient numbers (N > 200)
DOE-A / DUE-A: Differentially over- or under-expressed genesets from the entire twelve datasets with any patient numbers
*CCNA2*: Cyclin A2
*CENPN*: Centromere protein N
*DEPDC1*: DEP domain containing 1
*TTK*: TTK protein kinase (Thr/Tyr kinase)
*ABAT*: 4-Aminobutyrate aminotransferase
*LRIG1*: Leucine-rich repeats and immunoglobulin like domains 1
*PGR*: Progesterone receptor
*IL6*: Interleukin 6
*CXCL8*: C-X-C motif chemokine ligand 8
*AURKA*: Aurora kinase A
*NOTCH1*: Notch receptor 1
*CDC20*: Cell division cycle 20
*APOE*: Apolipoprotein E
*CDKN2A*: Cyclin-dependent kinase inhibitor 2A
*KIF2C*: Kinesin family member 2C
*ESR1*: Estrogen receptor 1
*FOXA1*: Forkhead box A1
*GATA3*: GATA binding protein 3
*EEF2*: Eukaryotic translation elongation factor 2
*RPL7A*: Ribosomal protein L7a
*TFF1*: Trefoil factor 1
*RPL15*: Ribosomal protein L15
*AR*: Androgen receptor
*IGF1R*: Insulin-like growth factor 1 receptor
STRING: Search tool for the retrieval of interacting genes
DC: Degree of connectivity
CCLE: Cancer Cell Line Encyclopedia
BL: Basal-like
TNBC: Triple-negative breast cancer
PAM50: Prediction analysis of microarray 50 (PAM50)
IHC: Immunohistochemistry
KM plotter: Kaplan-Meier plotter
RFS: Relapse-free survival
OS: Overall survival
DMFS: Distant metastasis-free survival (DMFS)
PPS: Post-progression survival
MRFS: Metastatic relapse-free survival
HR: Hazardous ratio
CI: Confidence interval
BP: Biological process
MF: Molecular function
CC: Cellular component
hub_oe: Hub genes co-expressed with DOE-A genes
hub_ue: Hub genes co-expressed with DUE-A genes
TCGA: The Cancer Genome Atlas
