# Supplementary figures and images for: In silico analysis of differentially expressed genesets in metastatic breast cancer identifies potential prognostic biomarkers
Source: World J Surg Oncol. 2021 Jun 25;19:188. doi: 10.1186/s12957-021-02301-7 (PMC8235641; doi:10.1186/s12957-021-02301-7)

# Supplementary Figure S1

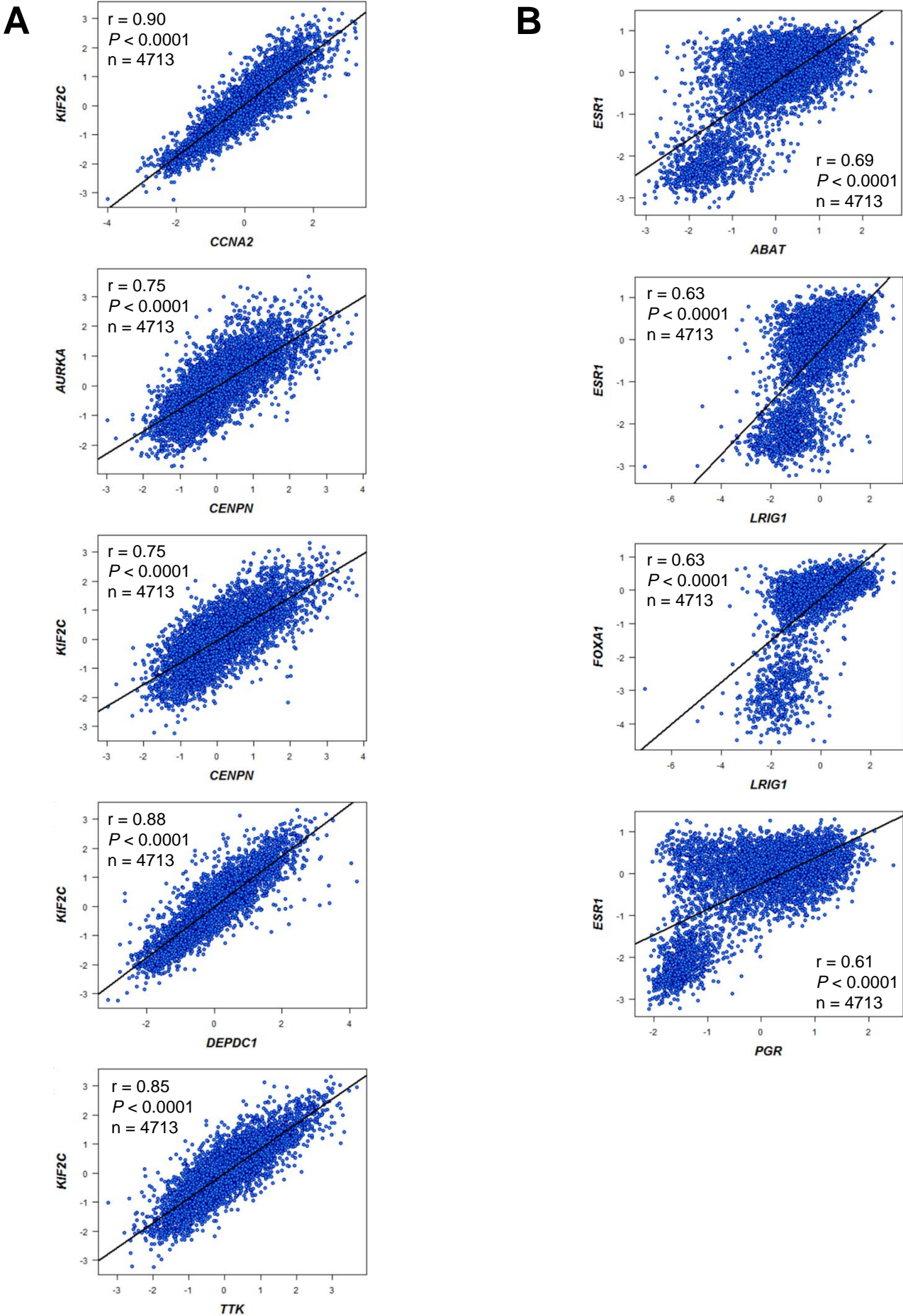

# Supplementary Figure S2

A

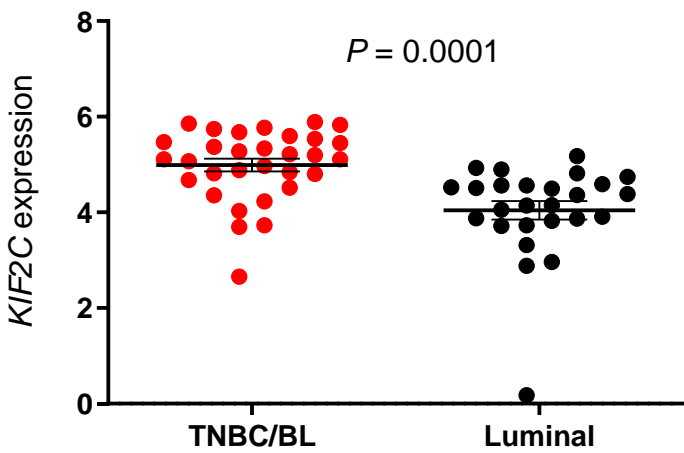

B

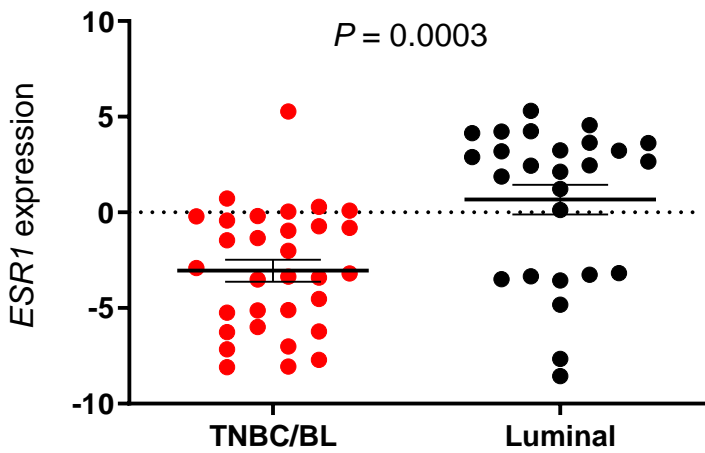

C

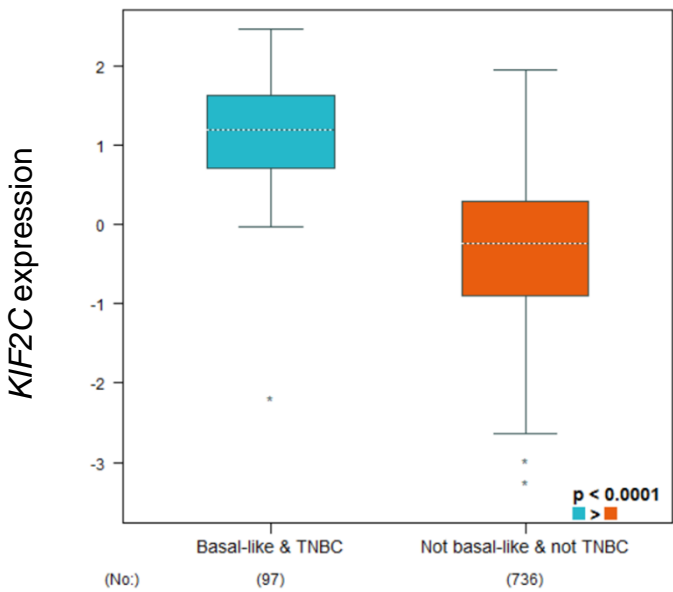

D

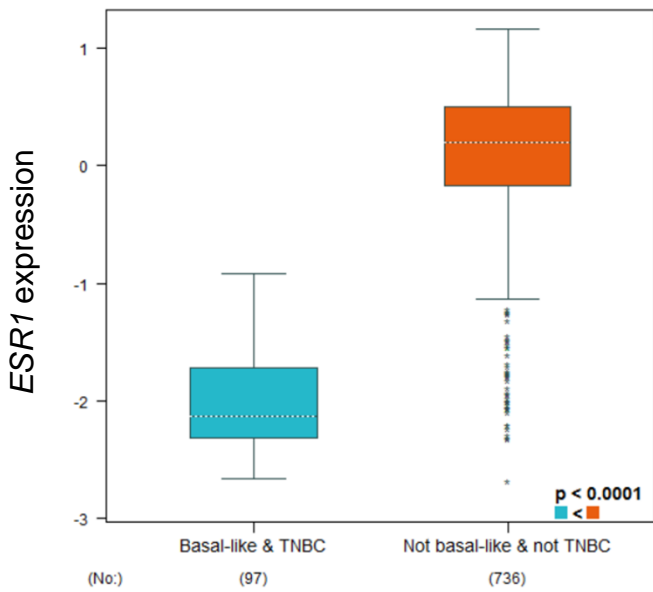

# Supplementary Figure S3

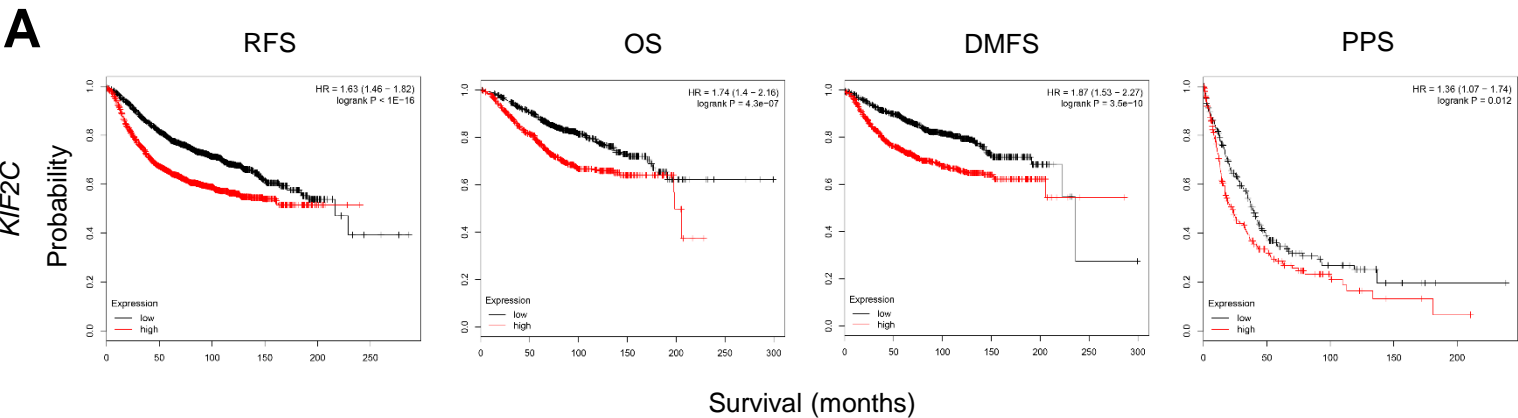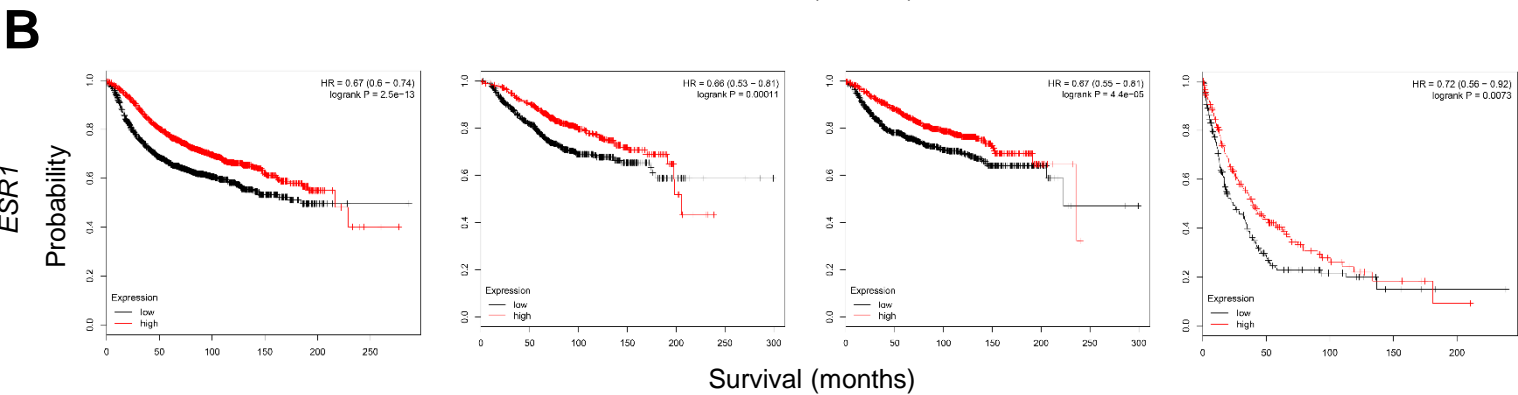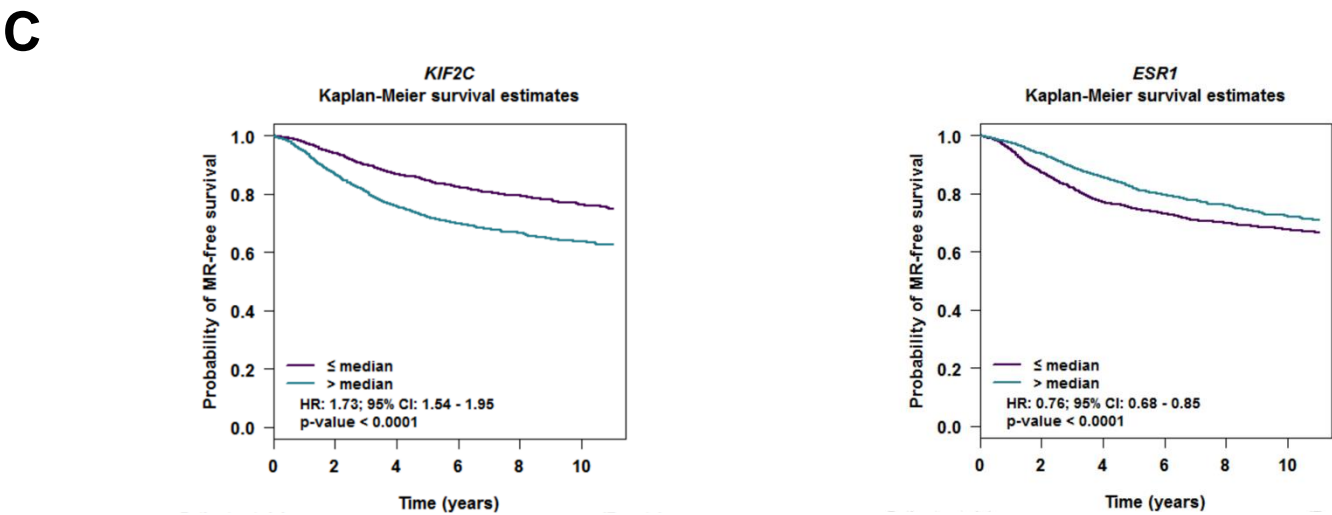

Supplement: Supplementary file 1 — Additional file 1: Supplementary Figure S1. KIF2C and ESR1 are hub genes the most significantly co-expressed with the potential biomarker candidate genes. (A and B) Shown are Pearson's pairwise correlation plots of RNA-seq gene expression between four DOE-A (A) or three DUE-A genes (B) and their most significantly co-expressed hub genes identified from PPI networks. Statistical analyses were performed by the pre-set analytic method of bc-GenExMiner. Supplementary Figure S2. Comparison of mRNA expression of the two most significantly co-expressed hub genes (KIF2C and ESR1) between basal-like or triple-negative breast cancer and other subtypes of breast cancer. (A and B) RNA-seq data of KIF2C and ESR1 were obtained from the Cancer Cell Line Encyclopedia (CCLE) and analyzed. N = 31 in BL/TNBC and N = 26 in luminal type cell lines. (C and D) RNA-seq data of KIF2C and ESR1 from The Cancer Genome Atlas (TCGA) [58] were analyzed at bc-GenExMiner v4.3. N = 97 in BL/TNBC and N = 736 in non-BL/TNBC type breast cancer patient samples. Statistical significance in A and B was determined by unpaired t-tests and those in C and D were determined by the pre-set analytic method of bc-GenExMiner. Supplementary Figure S3. Correlation between the expression levels of two co-expressed hub genes (KIF2C and ESR1) and patient survivals. (A and B) Relapse-free, overall, distant metastasis-free, and post-progression survival of two co-expressed hub genes (KIF2C in (A); ESR1 in (B)) were stratified by the expression levels of each gene (low or high). Expression data were analyzed by KM plotter (http://kmplot.com/). JetSet best probes were selected and patients (for KIF2C, N = 3951 in RFS, = 1402 in OS, = 1746 in DMFS and = 414 in PPS; for ESR1, N = 3951 in RFS, = 1402 in OS, = 1746 in DMFS and = 414 in PPS) were split by median expression. (C) Metastatic relapse-free survival of KIF2C and ESR1 was stratified by the expression levels of each gene (low or high). Microarray expression data were a [file 12957_2021_2301_MOESM1_ESM.zip › Suppl. Figs1-3.pdf]
